# Cancer mortality in Serbia, 1991–2015: an age-period-cohort and joinpoint regression analysis

**DOI:** 10.1186/s40880-018-0282-3

**Published:** 2018-04-10

**Authors:** Milena Ilic, Irena Ilic

**Affiliations:** 1 Department of Epidemiology, Faculty of Medical Sciences, University of Kragujevac, S. Markovica 69, Kragujevac, 34000 Serbia; 2 Faculty of Medicine, University of Belgrade, Belgrade, 11000 Serbia

**Keywords:** Cancer, Mortality, Trend, Joinpoint regression analysis, Age-period-cohort analysis

## Abstract

**Background:**

As the result of dramatic political changes, civil wars, and a long-term refugee crisis from the end of the last to beginning of this century, the population of Serbia has experienced significant health problems. The aim of this study was to assess cancer mortality trends in Serbia.

**Methods:**

This nationwide study was carried out to analyze cancer mortality in Serbia during 1991–2015 using official data. The age-standardized mortality rates (per 100,000) were calculated by direct standardization, using the world standard population by Segi. The average annual percent change (AAPC) and corresponding 95% confidence interval (CI) were computed using joinpoint regression analysis. Age-period-cohort analysis was performed to address the possible underlying reasons for the observed temporal trends.

**Results:**

Over the 25-year study period, there were 466,075 cancer deaths (266,043 males and 200,032 females) in Serbia. Overall cancer mortality increased between 1991 and 2009 in both males (by + 0.9% per year) and females (by + 0.8% per year) and has been decreasing since then, by − 0.9% annually in both sexes. For almost all major cancers except stomach cancer, cancer mortality in Serbia demonstrated upward trends during the study period. The largest increases were noted in lung cancer among females (AAPC = + 3.7, 95% CI 3.5–3.9) and prostate cancer in males (AAPC = + 1.9, 95% CI 1.4–2.3).

**Conclusions:**

After two decades of increase, cancer mortality rates are finally declining in Serbia. Despite this, these rates place Serbia among the countries with the highest cancer mortality in the world.

## Introduction

In recent decades, cancer has become one of the leading causes of death worldwide in both sexes, with significant geographic variations in frequency and distribution [1–3]. Based on GLOBOCAN 2012 estimates, overall cancer mortality in Serbia (147.8 per 100,000) ranks among the highest in the world after Mongolia (161.0 per 100,000), Hungary (152.1 per 100,000), and Armenia (150.3 per 100,000) [2]. The lowest mortality rates have been recorded in countries of South-Central Asia and Western Africa (approximately 70.0 per 100,000).

Since the late 1980s, cancer mortality rates have been declining in most developed countries [4–7]. In contrast, cancer mortality trends in some Eastern and Southern European countries have risen continuously [7, 8]. It is considered that the decrease in overall cancer mortality rates in developed countries is attributable to the reduction in tobacco smoking-related cancers (i.e., lung, intestinal, prostate, and cervical cancers, among others) [3, 5, 7]. The reduction in cancer mortality in Western countries might also be attributed to reductions in other major risk factors (e.g., dietary habits, alcohol use, occupational exposure to carcinogens, infection, and so on) as well as the availability and use of screening, early diagnosis, and improved treatment. The increased cancer mortality in developing countries probably reflects lifestyle changes that accompany industrialization, including greater consumption of animal fats, increased obesity, and physical inactivity [8, 9].

Serbia (officially the Republic of Serbia) is a small country in southeastern Europe, where the previous 25 years have been marked by dramatic socioeconomic and political upheaval, such as civil wars from 1991 to 1999, the break-up of the former Yugoslavia, influx of more than a million refugees, damaging effects of economic sanctions imposed by the United Nations from 1992 to 1995, a 78-day bombing campaign by NATO (North Atlantic Treaty Organization) in 1999, the collapse of the economy, decline of the living standard of the population, degradation of health services (with a lack of drugs and other medical materials), democratic changes in social and political circumstances in 2000, and the global financial crisis in 2007. With democratic political changes since 2000, there have been some general improvements of the social conditions in Serbia. Despite some enhancements of the health system, however, the Serbian population continues to experience significant health problems [10]. As yet, there are no corresponding data for cancer mortality in the Serbian population for that period.

In this nationwide study, we assessed cancer mortality trends in Serbia over a 25-year period, which includes the time of civil war in the country and the global economic crisis. In addition, we obtained comprehensive estimates of the nationwide cancer mortality pattern in Serbia.

## Materials and methods

### Data sources

For this nationwide study, we used data of annual underlying mortality causes from Serbia to describe cancer mortality trends for the period 1991–2015.

Data on individuals who died of cancer [International Classification of Diseases (ICD) Ninth Revision codes 140–209 and Tenth Revision codes C00–C97 to classify death, injury, and cause of death] were obtained from the Statistical Office of the Republic of Serbia. Sites for analysis included overall cancer and the 13 most common cancers: stomach (C16), colon and rectum (“colon”, C18–C21), pancreas (C25), lung and bronchus (“lung”, C33–C34), breast (C50), cervix uteri (C53), ovary (C56), prostate (C61), urinary bladder (C67), kidney (C64–C65), meninges, brain and central nervous system (“brain”, C70–C72), and malignant lymphoma and leukemia (“hematological malignancies”, C81–C96).

This research included the entire population of the Republic of Serbia (all ages), excluding the Autonomous Province of Kosovo and Metohija. The Statistical Office of the Republic of Serbia does not have data relative to the Autonomous Province Kosovo and Metohija, for which data are unavailable from 1998 onward and which declared itself independent in 2008; as such, these data could not be included in the coverage for the Republic of Serbia.

National mortality statistics data were reported on two forms: Death Certificate (statistical form for reporting deaths, Form DEM-2) and Physician’s Certificate of Cause of Death. The sources of data were health institutions and local death registers. The procedure comprised several levels of control and verification. Specifically, the local registrar reviewed and forwarded death files to the referral public health institute, where a trained medical doctor or specialist checked the file against the death certificate. Authorized medical doctors extracted the underlying cause of death from the death certificate and entered the appropriate codes for the underlying cause of death into Form DEM-2.

The data relating to mortality in Serbia are reliable [11]. The completeness of the Serbian mortality database was 100% in 2000, and the coverage was 97%. In addition, the mortality/incidence ratio (about 60%) indicated that completeness of the mortality database was satisfactory. Validity of the mortality data was confirmed through the percentage of microscopically (histologically) verified new cancers (77.1% in males and 82.6% in females in Central Serbia only) and the percentage of cancers certified only after death (2.9% in males and 2.6% in females in Central Serbia only). The data are also comparable with those of different countries and over time. Serbia is currently using the 10th revision of the ICD (ICD-10); the ICD-9 was used from 1991 to 1996. The proportion of deaths assigned ICD codes for “symptoms, signs, and ill-defined conditions” (ICD-9 codes 780–799 and ICD-10 codes R00–R99) was 8% in 2000. The World Health Organization classifies Serbian death registration data as medium quality.

### National population estimates

Data on the number and composition (by sex and age) of the population of the Republic of Serbia were obtained from the population censuses in the years 1991, 2002, and 2011. For inter-census years, the estimates published by the Statistical Office of the Republic of Serbia were used. In the observed period, the Serbian population continuously declined, from 7,600,000 inhabitants in 1991 to 7,100,000 inhabitants in 2015.

### Statistical analysis

For the entire Serbian population, we calculated three types of cancer mortality rates: crude, specific (including age-specific, sex-specific, and cause-specific) and standardized mortality rates. Mortality rates were expressed per 100,000 persons. Calculations were based on the 10-year age group in which the cancer death occurred. The age-standardized rates (ASRs) were calculated by direct standardization, using the world standard population, as proposed by Segi [12].

Cancer mortality trends were estimated using joinpoint regression analysis. The Joinpoint Regression Program (version 3.5.3, released May 2013) is trend analysis software developed by the United States National Cancer Institute (US NCI) for the analysis of data from the Surveillance Epidemiology and End Results Program (SEER), according to the method proposed by Kim et al. [13]. Joinpoint analysis was used to identify the best-fitting point, where a statistically significant change (called the “joinpoint”) had occurred, and to determine the trends between joinpoints. The “grid search” method was selected [14]. The tests of significance used included the Monte Carlo permutation method with 4499 randomly selected datasets, to determine the best-fitting combination of line segments and joinpoints [13]. The number of joinpoints ranged from “0” to “5”. Cancer mortality trends are measured using the annual percentage change (APC) between successive change points, and the corresponding 95% confidence interval (CI) [15]. In the final model, the joinpoint analysis also provides an average annual percentage change (AAPC) as a trend analysis summary measure. Disparities in cancer mortality trends by sex and age were tested using comparability testing, to determine whether the two regression mean functions were identical (test of coincidence) or parallel (test of parallelism) [16]. Two-sided *p* values less than 0.05 were considered to indicate statistical significance.

Additionally, we performed age-period-cohort analysis to examine the effects of age, period, and birth cohort on the observed temporal trends using the US NCI web-based statistical tool, according to the method proposed by Rosenberg et al. [17]. In the age-period-cohort analysis, we used the overall cancer mortality data by consecutive 5-year age groups (0–4, …, and 80–84), and the same 5-year intervals for calendar periods (1991–1995, 1996–2000, … and 2011–2015) and birth cohorts (1911–1915, …, and 2011–2015). The parameters of the age-period-cohort analysis included longitudinal age-specific rates, period and cohort rate ratios, and local drifts with net drift. Longitudinal age curves indicated the fitted longitudinal age-specific rates in the reference cohort, adjusted for period deviations. The period effects represent variations in mortality rates over time associated with all age groups simultaneously. The cohort effects are associated with changes in mortality rates across groups of individuals with the same birth years, that is, for successive age groups in successive time periods. The “local drifts”, which represent the annual percentage changes for each age group, were generated from log-linear regressions. The “net drift”, which represents the average annual percentage change in mortality per year of birth (a quantity that cannot be attributed specifically to the cohort), in fact was the sum of the log-linear temporal trend arising from birth cohort effects. The significance test used was a 1 − *df* Wald test. *p* values less than 0.05 were considered statistically significant. Considering that there were very few cases in the age groups less than 30 years or more than 85 years, with consequently unstable mortality rates, we omitted these age groups from the age-period-cohort analysis for particular cancers, by sex.

## Results

### Cancer mortality rates

Over the 25-year study period, there were 466,075 cancer deaths (266,043 males and 200,032 females), with overall average annual ASR of 132.9 per 100,000 (ASRs ranged from 120.6 per 100,000 in 1991 to 142.3 per 100,000 in 2010) (Table 1). Average annual ASRs were 167.7/100,000 for males and 104.8/100,000 for females.

**Table 1:** Cancer mortality in Serbia during 1991–2015, by sex

Year	Males			Females			Total		
	Number of deaths	Crude mortality (/100,000)	ASR of mortality (/100,000)	Number of deaths	Crude mortality (/100,000)	ASR of mortality (/100,000)	Number of deaths	Crude mortality (/100,000)	ASR of mortality (/100,000)
1991	8291	223.1	152.4	6263	161.4	95.0	14,554	191.6	120.6
1992	8784	236.3	158.6	6526	167.9	98.4	15,310	201.3	124.9
1993	8835	237.4	157.3	6552	168.3	97.5	15,387	202.1	124.1
1994	8777	235.7	153.5	6598	169.2	96.7	15,375	201.7	121.9
1995	8965	240.6	154.9	6895	176.7	99.2	15,860	207.9	124.2
1996	9359	251.4	159.1	7001	179.6	100.4	16,360	214.6	126.4
1997	9671	260.8	162.4	7296	187.5	102.8	16,967	223,3	129.2
1998	9756	264.6	162.3	7423	191.3	102.8	17,179	227.0	129.3
1999	9869	269.0	162.9	7506	193.9	102.6	17,375	230.4	129.5
2000	10,178	278.4	165.7	7694	199.3	104.3	17,872	237.8	131.9
2001	10,173	278.8	164.3	7742	200.8	103.5	17,915	238.8	130.6
2002	10,634	291.6	169.2	7913	205.4	105.2	18,547	247.3	133.6
2003	10,688	293.8	169.2	8169	212.6	106.4	18,857	252.1	134.5
2004	10,980	302.5	171.4	8378	218.5	107.8	19,358	259.4	136.3
2005	11,166	308.6	174.2	8571	224.2	108.8	19,737	265.3	138.1
2006	11,494	319.0	177.0	8722	229.1	110.5	20,216	272.8	140.2
2007	11,730	326.8	178.7	8681	228.9	108.4	20,411	276.5	139.8
2008	11,807	330.4	177.9	8763	232.0	110.3	20,570	279.9	140.4
2009	11,982	336.6	179.2	9048	240.6	111.7	21,030	287.3	141.8
2010	12,113	341.6	180.4	9018	240.8	111.4	21,138	289.9	142.3
2011	12,085	342.9	174.4	8922	240.3	107.5	21,007	290.3	137.3
2012	12,133	346.0	173.2	9136	247.3	109.3	21,262	295.3	137.7
2013	12,101	346.8	171.5	8990	244.5	106.5	21,091	294.3	135.3
2014	12,095	348.3	170.4	9227	252.2	108.4	21,322	299.0	135.9
2015	12,377	357.3	173.4	8998	246.5	105.5	21,375	300.4	135.7
Overall	266,043	294.7	167.7	200,032	210.4	104.8	466,075	252.6	132.9

*ASR* age standardized rate (using world standard population by Segi)

### Cancer mortality trends: joinpoint analysis

**Fig. 1:**
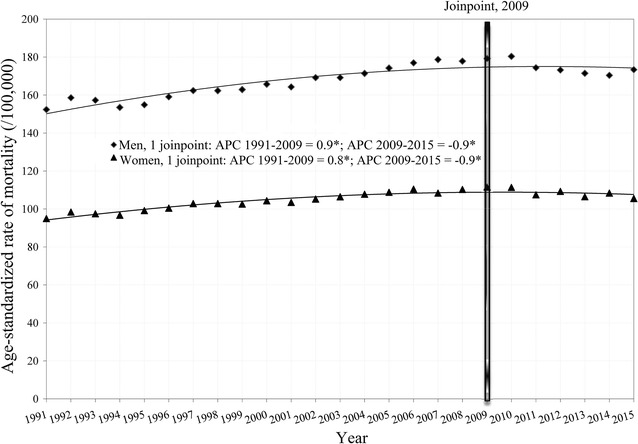
Joinpoint regression analysis of cancer mortality trends in Serbia, by sex, during 1991–2015. *statistically significant trend. Age-standardized rate (/100,000, using world standard population). *APC* annual percent change

We found the cancer mortality trend to be significantly increased from 1991 to 2009 in both the male (APC = + 0.9, 95% CI 0.8–1.0) and female (APC = + 0.8, 95% CI 0.7–0.9) populations (Fig. 1). Since 2009, the overall cancer mortality trend has significantly decreased (by − 0.9% yearly in both sexes). According to the comparability test, cancer mortality trends in males and females were parallel (*p *= 0.624).

Death rates from all major cancers were higher among males than among females in Serbia (Tables 2 and 3). All major cancers accounted for 79% of overall cancer mortality. The most common cancers in males were lung (31.0% of all cancer deaths in males), colon (11.8%), prostate (7.1%), stomach (6.9%), and hematological malignancies (5.1%) whereas those in females were breast (18.4%), lung (12.2%), colon (11.6%), cervix uteri (5.6%), and hematological malignancies (5.3%).

**Table 2:** Cancer mortality among males in Serbia, 1991–2015

Number of cancer deaths and mortality						
5-years of report						
Cancer site	1991–1995	1996–2000	2001–2005	2006–2010	2011–2015	Total
Lung						
Deaths	13,221	15,178	16,539	18,681	18,784	82,401
ASR	46.8	50.1	52.8	58.4	55.1	52.6
Colon						
Deaths	4428	5488	6326	7313	7874	31,429
ASR	15.5	17.6	19.2	20.8	21.1	18.8
Stomach						
Deaths	3826	3921	3871	3497	3167	18,282
ASR	13.3	12.7	11.7	10.3	8.7	11.4
Hematological malignancies						
Deaths	2309	2237	2623	3143	3261	13,573
ASR	9.2	8.1	9.1	10.1	9.9	9.3
Brain						
Deaths	1328	1538	1744	1945	1948	8504
ASR	5.3	6.0	6.5	6.9	6.5	6.2
Bladder						
Deaths	1545	1734	2007	2219	2548	10,053
ASR	5.3	5.5	5.8	5.9	6.5	5.8
Pancreas						
Deaths	1803	2063	2356	2684	2920	11,826
ASR	6.3	6.8	7.3	8.1	8.3	7.3
Prostate						
Deaths	2506	2806	3669	4826	5118	18,925
ASR	8.7	8.7	10.2	11.9	11.9	10.3
Renal						
Deaths	813	883	998	1142	1267	5103
ASR	2.9	3.0	3.2	3.6	3.7	3.3
All sites						
Deaths	43,652	48,833	53,641	59,126	60,791	266,043
ASR	155.3	162.5	169.7	178.6	172.6	167.7

*ASR* age standardized rate (/100,000, using world standard population)

**Table 3:** Cancer mortality among females in Serbia, 1991–2015

Number of cancer deaths and mortality						
5-years of report						
Cancer site	1991–1995	1996–2000	2001–2005	2006–2010	2011–2015	Total
Lung						
Deaths	3011	3703	4695	6000	6974	24,383
ASR	8.8	10.3	12.6	15.8	17.7	13.0
Breast						
Deaths	6047	6880	7459	8047	8297	36,730
ASR	18.9	20.5	20.9	21.1	20.2	20.3
Colon						
Deaths	3660	4264	4772	5293	5206	23,195
ASR	10.0	10.9	11.2	11.8	11.1	11.0
Stomach						
Deaths	2123	2239	2213	2020	1810	10,405
ASR	6.0	5.8	5.3	4.7	4.0	5.2
Hematological malignancies						
Deaths	1693	1802	2062	2477	2478	10,512
ASR	5.7	5.5	5.8	6.5	6.2	6.0
Cervix uteri						
Deaths	2069	2197	2321	2458	2235	11,280
ASR	6.5	6.8	7.0	7.3	6.5	6.8
Ovary						
Deaths	1552	1537	1720	2001	2160	8970
ASR	4.8	4.6	4.8	5.5	5.5	5.0
Brain						
Deaths	974	1198	1357	1590	1534	6653
ASR	3.5	4.1	4.4	4.9	4.3	4.3
Bladder						
Deaths	483	582	618	723	808	3214
ASR	1.2	1.4	1.3	1.4	1.5	1.4
Pancreas						
Deaths	1352	1666	1946	2334	2496	9794
ASR	3.7	4.2	4.6	5.1	5.4	4.6
Renal						
Deaths	594	652	658	687	717	3308
ASR	1.7	1.7	1.6	1.5	1.6	1.6
All sites						
Deaths	32,834	36,920	40,773	44,232	45,273	200,032
ASR	97.4	102.6	106.3	110.5	107.4	104.8

*ASR* age standardized rate (/100,000, using world standard population)

In both sexes, cancer mortality has significantly decreased since 1991 in all age groups under 40 years, except in females aged 0–9 years where a non-significant decrease was present (Table 4). In addition, the cancer mortality trends showed a significant increase in males and females in all age groups 50 years and above.

**Table 4:** Joinpoint analysis: trends* in age-specific cancer mortality rates (per 100,000 persons) among males and females in Serbia, 1991–2015

Age	Males		Females	
	Period	APC (95% CI)	Period	APC (95% CI)
0–9	1991–2015	− 2.3* (− 3.9 to − 0.6)	1991–2015	− 1.4 (− 2.8 to 0.0)
10–19	1991–2015	− 3.5* (− 5.2 to − 1.8)	1991–2015	− 2.2* (− 4.1 to − 0.2)
20–29	1991–2015	− 1.6* (− 2.5 to − 0.8)	1991–2015	− 1.3* (− 2.4 to − 0.2)
30–39	1991–2015	− 1.6* (− 2.2 to − 1.0)	1991–2015	− 1.6* (− 1.8 to − 1.3)
40–49	1991–1998	2.3* (1.2 to 3.4)	1991–2002	0.9* (0.0 to 1.8)
	1998–2008	− 1.7* (− 2.5 to − 1.0)	2002–2015	− 1.9* (− 2.6 to − 1.2)
	2008–2015	− 4.2* (− 5.2 to − 3.2)	AAPC	− 0.7* (− 1.1 to − 0.3)
	AAPC	− 1.4* (− 1.9 to − 0.9)		
50–59	1991–1994	− 1.2 (− 3.7 to 1.3)	1991–2008	1.4* (1.0 to 1.8)
	1994–2007	1.9* (1.5 to 2.2)	2008–2015	− 1.2 (− 2.5 to 0.3)
	2007–2015	− 2.3* (− 2.8 to − 1.7)	AAPC	0.8* (0.6 to 1.1)
	AAPC	0.6* (0.2 to 0.9)		
60–69	1991–2015	0.7* (0.5 to 0.8)	1991–2015	0.8* (0.7 to 1.0)
70–79	1991–2009	1.5* (1.2 to 1.8)	1991–2009	1.2* (1.0 to 1.4)
	2009–2015	− 0.6 (− 2.1 to 0.9)	2009–2015	− 0.9 (− 1.9 to 0.0)
	AAPC	1.1* (0.9 to 1.4)	AAPC	0.8* (0.6 to 1.0)
80+	1991–1994	− 2.5 (− 8.4 to 3.7)	1991–2015	1.4* (1.2 to 1.7)
	1994–2015	2.6* (2.3 to 2.9)		
	AAPC	2.3* (2.0 to 2.6)		

AAPC (average annual percent change) presented for full period

*APC* annual percent change; *CI* confidence interval

^*^ Statistically significant trend

For almost all major cancers in Serbia except stomach cancer, cancer mortality demonstrated upward trends in both sexes during the study period (Tables 5 and 6). Mortality rates from all major cancers in Serbia were higher in males than in females. The largest increases were in lung cancer for females (AAPC = + 3.7, 95% CI 3.5–3.9) and prostate cancer for males (AAPC = + 1.9, 95% CI 1.4–2.3). The upward trend in lung cancer mortality was much greater for females than for males (+ 3.5% versus + 1.0% per year, respectively). Bladder cancer was the only cancer for which an upward trend (by + 0.9% per year) was seen in both sexes equally and continuously during the entire studied period. The largest increase was in mortality for brain cancer in both sexes: by + 8.5% (95% CI 1.6–15.9) per year from 1991 to 1995 in females, and by + 17.8% (95% CI − 5.9–47.6) per year from 1991 to 1993 in males.

**Table 5:** Time trends of age-standardized mortality rates from common cancers among males in Serbia, 1991–2015, by joinpoint analysis

Cancer site	AAPC (95% CI)	Trend 1	APC (95% CI)	Trend 2	APC (95% CI)	Trend 3	APC (95% CI)
Lung	1.0* (0.7 to 1.2)	1991–2009	1.4* (1.1 to 1.6)	2009–2015	− 1.1 (− 2.4 to 0.2)	None	–
Colon	1.6* (1.3 to 1.8)	1991–2009	2.0* (1.7 to 2.3)	2009–2015	− 0.3 (− 1.9 to 1.2)	None	–
Stomach	− 2.1* (− 2.4 to − 1.8)	1991–2009	− 1.6* (− 1.9 to − 1.3)	2009–2015	− 4.6* (− 6.2 to − 2.9)	None	–
Hematological malignancies	0.7* (0.3 to 1.1)	1991–1998	− 3.0* (− 4.2 to − 1.9)	1998–2007	3.0* (2.0 to 4.0)	2007–2015	− 0.4 (− 1.4 to 0.6)
Brain	1.2* (0.6 to 1.7)	1991–1993	17.8 (− 5.9 to 47.6)	1993–2015	0.8* (0.2 to 1.3)	None	–
Bladder	0.9* (0.4 to 1.3)	None	–	None	–	None	–
Pancreas	1.4* (1.1 to 1.7)	1991–1993	− 5.6 (− 17.5 to 1.3)	1993–2015	1.6* (1.3 to 1.9)	None	–
Prostate	1.9* (1.4 to 2.3)	None	–	None	–	None	–
Renal	1.4* (0.9 to 1.8)	None	–	None	–	None	–
All sites	0.6* (0.5 to 0.8)	1991–2009	0.9* (0.8 to 1.0)	2009–2015	− 0.9* (− 1.6 to − 0.2)	None	–

*AAPC* average annual percent change; *APC* annual percent change; *CI* confidence interval

^*^ Statistically significant trend

**Table 6:** Time trends of age-standardized mortality rates from common cancers among females in Serbia, 1991–2015, by joinpoint analysis

Cancer site	AAPC (95% CI)	Trend 1	APC (95% CI)	Trend 2	APC (95% CI)	Trend 3	APC (95% CI)
Lung	3.7* (3.5 to 3.9)	1991–2010	4.0* (3.7 to 4.3)	2010–2015	1.9 (− 0.3 to 4.1)	None	–
Breast	0.3* (0.1 to 0.6)	1991–2004	1.1* (0.6 to 1.6)	2004–2015	− 0.6* (− 1.2 to 0.0)	None	–
Colon	0.5* (0.2 to 0.9)	1991–2009	1.1* (0.6 to 1.5)	2009–2015	− 2.0 (− 4.3 to 0.3)	None	–
Stomach	− 2.0* (− 2.4 to − 1.6)	None	–	None	–	None	–
Hematological malignancies	0.6* (0.2 to 1.0)	None	–	None	–	None	–
Cervix uteri	0.1 (-0.3 to 0.5)	1991–2008	0.9* (0.4 to 1.3)	2008–2015	− 2.7* (− 4.4 to − 0.9)	None	
Ovary	0.9* (0.6 to 1.3)	None	–	None	–	None	–
Brain	1.1* (0.4 to 1.8)	1991–1995	8.5* (1.6 to 15.9)	1995–2010	1.2* (0.2 to 2.2)	2010–2015	− 4.1 (− 8.5 to 0.4)
Bladder	0.9* (0.5 to 1.3)	None	–	None	–	None	–
Pancreas	1.9* (1.6 to 2.3)	None	–	None	–	None	–
Renal	-0.4 (-0.9 to 0.2)	None	–	None	–	None	–
All sites	0.5* (0.4 to 0.7)	1991–2009	0.8* (0.7 to 0.9)	2009–2015	− 0.9* (− 1.5 to − 0.4)	None	–

*AAPC* average annual percent change; *AP*C annual percent change; CI confidence interval

^*^ Statistically significant trend

### Cancer mortality trends: age-period-cohort analysis

**Fig. 2:**
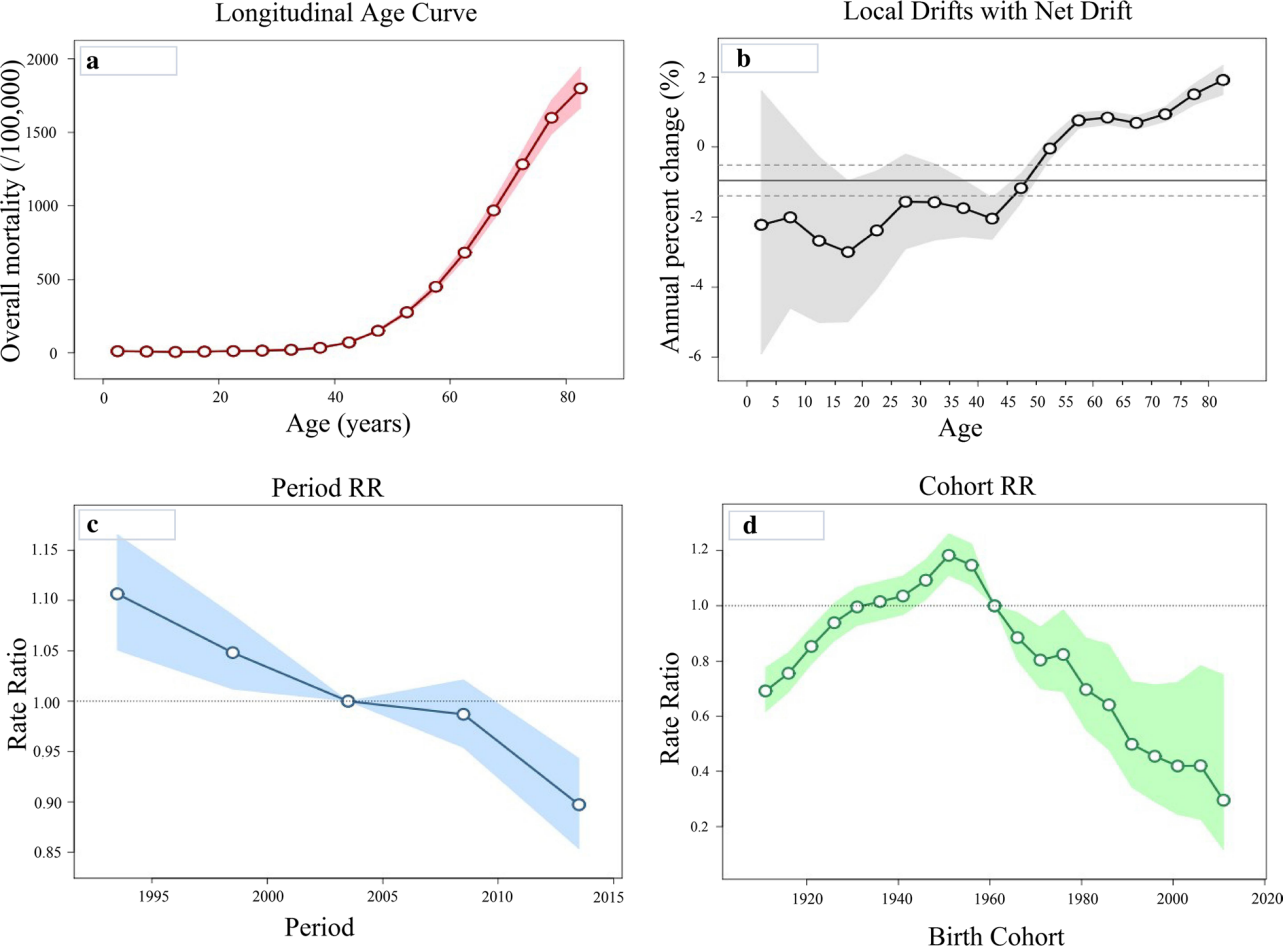
Cancer mortality among males in Serbia during 1991–2015, by age-period-cohort analysis. **a** Longitudinal age curve of cancer mortality rates (per 100,000 people) and corresponding 95% confidence intervals (pink area). Expected age-specific rates in reference cohort c0 adjusted for period effects: the risk of cancer increased with age in males. **b** Local drift value for cancer mortality rates: age group-specific annual percent change (%) in cancer mortality rates and corresponding 95% confidence intervals (grey area). Annual percentage change of the expected age-specific rates over time: the local drifts and net drift were statistically significant (*p *< 0.05). **c** Period effects on cancer mortality rates: obtained from age-period-cohort analyses for cancer mortality rates and corresponding 95% confidence intervals (blue area). Period rate ratios (RRs) are illustrated for cancer mortality relative to the 2003 reference year. The horizontal line indicates RR of 1 (no difference between a selected period and the reference year). Ratio of age-specific rates in each period relative to reference period p0: period effect was statistically significant for males (*p *< 0.05). **d** Cohort effects on cancer mortality rates: obtained from age-period-cohort analyses for cancer mortality rates and corresponding 95% confidence intervals (green area*)*. Cohort rate ratios (RRs) are illustrated for cancer mortality relative to the 1961 reference birth cohort. The horizontal line indicates RR of 1 (no difference between a selected birth cohort and the reference cohort). Ratio of age-specific rates in each cohort relative to reference cohort c0: cohort effect was statistically significant for males (*p *< 0.05)

Local drift values were under 0 in all age groups below 55 years in males, with significantly elevated local drift values at ages 55–84 years (Fig. 2). The period effects have showed downward trends since 1991. The cohort effects increased from 1911 to 1960 (although most of these values were under 1, with only a few significant exceptions for the cohorts from 1946 to 1960), with general downward trends from that point onwards. The Wald test indicated statistically significant cohort and period effects for males, as did the local drifts and net drift (Table 7).

**Table 7:** Age, period, and cohort effects on cancer mortality in Serbia during 1991–2015, by sex

Group	Men		Women	
	Effect	95% CI	Effect	95% CI
Age				
0–4	12.1	7.5–19.5	6.8	3.9–11.8
5–9	9.9	6.5–14.9	5.1	3.1–8.4
10–14	7.2	4.9–10.6	4.1	2.6–6.5
15–19	9.7	7.1–13.1	6.6	4.7–9.3
20–24	12.8	10.0–16.4	7.5	5.6–10.0
25–29	16.1	13.2–19.7	13.1	10.6–16.2
30–34	21.8	18.7–25.5	25.0	21.6–28.9
35–39	35.3	31.6–39.5	44.9	40.6–49.7
40–44	71.6	66.1–77.6	83.9	77.7–90.7
45–49	151.1	141.7–161.2	136.1	127.2–145.6
50–54	276.7	260.9–293.4	216.6	203.4–230.6
55–59	450.1	421.4–480.7	309.7	288.4–332.5
60–64	682.5	638.3–729.7	425.6	395.6–457.9
65–69	968.6	904.4–1037.4	582.4	540.1–628.0
70–74	1282.4	1195.7–1375.4	770.2	713.1–831.8
75–79	1599.0	1487.6–1718.7	985.3	910.2–1066.6
80–84	1798.6	1664.5–1943.5	1174.7	1080.0–1277.6
Period				
1991–1995	1.11	1.05–1.17	1.03	0.97–1.09
1996–2000	1.05	1.01–1.09	1.02	0.98–1.06
2001–2005	1.00	1.00–1.00	1.00	1.00–1.00
2006–2010	0.99	0.95–1.02	0.99	0.96–1.03
2011–2015	0.90	0.85–0.94	0.92	0.87–0.98
Cohort				
1911–1915	0.69	0.62–0.78	0.71	0.63–0.80
1916–1920	0.76	0.69–0.83	0.73	0.66–0.81
1921–1925	0.85	0.79–0.92	0.80	0.73–0.87
1926–1930	0.94	0.87–1.01	0.84	0.78–0.91
1931–1935	1.00	0.93–1.07	0.86	0.79–0.93
1936–1940	1.02	0.95–1.09	0.89	0.82–0.96
1941–1945	1.04	0.97–1.11	0.94	0.87–1.01
1946–1950	1.09	1.02–1.17	0.97	0.90–1.04
1951–1955	1.18	1.11–1.26	1.06	0.99–1.13
1956–1960	1.15	1.07–1.23	1.07	1.00–1.15
1961–1965	1.00	1.00–1.00	1.00	1.00–1.00
1966–1970	0.89	0.80–0.98	0.92	0.83–1.01
1971–1975	0.80	0.70–0.92	0.84	0.74–0.95
1976–1980	0.82	0.69–0.99	0.80	0.68–0.96
1981–1985	0.70	0.55–0.88	0.74	0.58–0.95
1986–1990	0.64	0.48–0.86	0.71	0.51–0.99
1991–1995	0.50	0.34–0.73	0.64	0.42–0.98
1996–2000	0.46	0.29–0.71	0.60	0.35–1.02
2001–2005	0.42	0.24–0.72	0.53	0.27–1.01
2006–2010	0.42	0.23–0.78	0.68	0.34–1.38
2011–2015	0.30	0.12–0.75	0.38	0.12–1.16
	Wald Chi square tests for estimable functions, *p* value			
Net drift	0.00			0.06
All period rate ratios	0.00			0.006
All cohort rate ratios	0.00			0.00
All local drifts	0.00			0.00

*CI* confidence interval

**Fig. 3:**
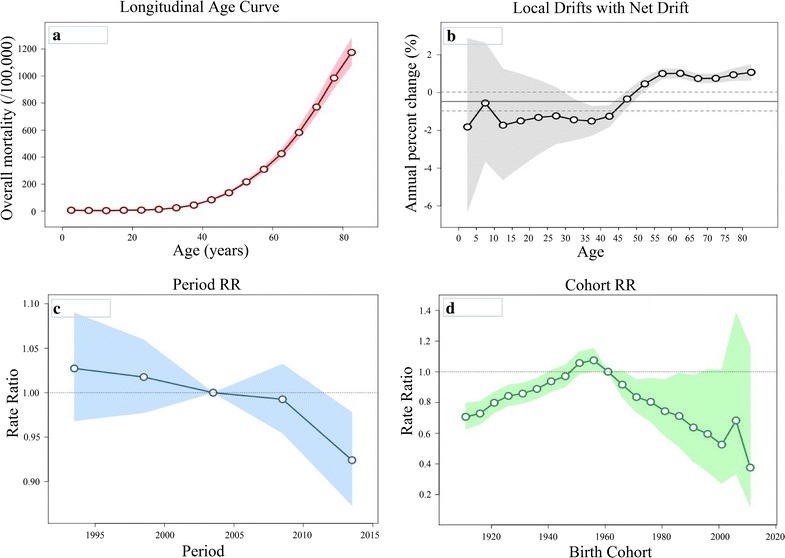
Cancer mortality among females in Serbia during 1991–2015, by age-period-cohort analysis. **a** Longitudinal age curve of cancer mortality rates (per 100,000 people) and corresponding 95% confidence intervals (pink area). Expected age-specific rates in reference cohort c0 adjusted for period effects: the risk of cancer increased with age in females. **b** Local drift value for cancer mortality rates: age group-specific annual percent change (%) in the cancer mortality rates and corresponding 95% confidence intervals (grey area). Annual percentage change of the expected age-specific rates over time: local drifts were statistically significant (*p *< 0.05) whereas net drift was not (*p *= 0.064). **c** Period effects on cancer mortality rates: obtained from age-period-cohort analyses for cancer mortality rates and corresponding 95% confidence intervals (blue area). Period rate ratios (RRs) are illustrated for cancer mortality relative to the 2003 reference year. The horizontal line indicates RR of 1 (no difference between a selected period and the reference year). Ratio of age-specific rates in each period relative to reference period p0: period effect was statistically significant for females (*p *= 0.006). **d** Cohort effects on cancer mortality rates: obtained from age-period-cohort analyses for the cancer mortality rates and corresponding 95% confidence intervals (green area). Cohort rate ratios (RRs) are illustrated for cancer mortality relative to the 1961 reference birth cohort. The horizontal line indicates RR of 1 (no difference between a selected birth cohort and the reference cohort). Ratio of age-specific rates in each cohort relative to reference cohort c0: cohort effect was statistically significant for females (*p *< 0.05)

The local drift values were under 0 in all age groups below 50 in females, with significantly elevated local drift values at ages 55–84 years (Fig. 3). The period effects have showed downward trends since 1991. The cohort effects increased from 1911 to 1960 (although most of these values were under 1, with only a few significant exceptions for the 1956–1960 cohort), with general downward trends from that point onwards. The Wald test indicated statistically significant cohort and period effects for females, as did the local drifts, whereas the net drift did not (Table 7).

The age-period-cohort analysis parameters for mortality of common cancers in males and females aged 30–84 years are displayed in Table 8. The age-period-cohort analysis estimated functions for cervical, ovarian, and colon cancers in females, as well as for bladder cancer in both sexes, were not significant. For mortality of brain cancers, Wald tests indicated statistically significant cohort and period effects for both sexes (*p *< 0.05 for all), as did the local drifts and net drift (*p *< 0.05 for all). For lung cancer mortality, statistically significant cohort and period effects were found for both sexes (*p *< 0.05 for all), as well as the local drifts (*p *< 0.05 for all); however, the net drift was significant only in females (*p *< 0.05). Statistically significant cohort effects were also noted for mortality of hematological malignancies and of colon, stomach, pancreas and prostate cancers in males; in females, statistically significant cohort effects were noted for mortality of hematological malignancies and of breast, stomach and pancreas cancers (*p *< 0.05 for all).

**Table 8:** Mortality from common cancers in males and females aged 30–84 years in Serbia, 1991–2015, by age-period-cohort analysis

	Lung	Breast	Colon	Stomach	Hematological malignancies	Pancreas	Brain	Prostate	Cervical	Ovarian	Bladder
Wald Chi square tests for estimable functions, *p* value											
Males											
Net drift	0.39		0.01	0.00	0.04	0.09	0.00	0.73			0.26
All period rate ratios	0.01		0.07	0.00	0.01	0.53	0.01	0.11			0.72
All cohort rate ratios	0.00		0.00	0.00	0.00	0.01	0.00	0.00			0.63
All local drifts	0.00		0.46	0.86	0.03	0.71	0.00	0.75			0.84
Females											
Net drift	0.00	0.92	0.30	0.00	0.06	0.02	0.00		0.97	0.05	0.13
All period rate ratios	0.00	0.91	0.17	0.00	0.12	0.17	0.03		0.39	0.08	0.55
All cohort rate ratios	0.00	0.00	0.88	0.00	0.00	0.01	0.00		0.66	0.07	0.93
All local drifts	0.00	0.00	0.98	1.00	0.19	1.00	0.00		0.42	0.71	0.91

**Fig. 4:**
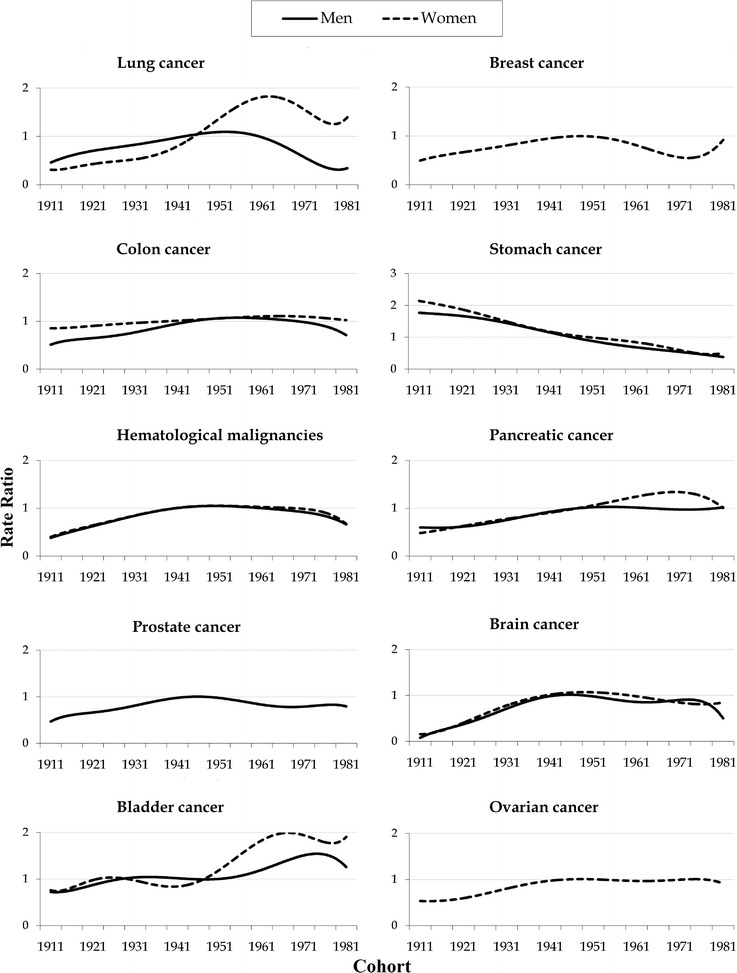
Cohort effects on mortality from common cancers among males and females aged 30–84 years in Serbia, 1991–2015, by age-period-cohort analysis. Rate ratio—cohort rate ratios are shown on relative risk scale

The risk of lung cancer mortality in Serbia increased with birth cohort in both sexes. The cohort effects remained stable for the first several birth cohorts, followed by an upward inflection for males born from 1951 to 1960 and for females born after 1951 (Fig. 4). In contrast to males, the risk for lung cancer by birth cohort in females shifted to a large increase since the 1951 cohort. There was no strong evidence for an effect of birth year on ovarian cancer mortality rates, and the increase in breast cancer mortality rates ended around 1955. A birth cohort effect on colon cancer mortality was seen in males from 1911 to 1940, but there was no evidence for the effect of birth year on colon cancer mortality rates in females. Prostate cancer mortality risk by birth cohort showed an increase from the 1911 up to the 1955 cohorts; the cohort trends were flat for the later cohorts. The cohort effects for pancreatic cancer mortality increased for the first several birth cohorts in both sexes, followed by an upward inflection in females born after 1950 and remaining stable in males. Mortality from hematological malignancies increased for the first several birth cohorts, followed by a downward inflection for people born after the 1960s. The pattern of brain cancer mortality by birth cohort for males and females was nearly identical, with risk increased for people born from 1911 to 1935; the cohort trends were flat for later cohorts.

## Discussion

Serbia’s mortality rates owing to cancer place it among the countries with the highest cancer mortality in the world. In the period from 1991 to 2015, approximately 266,000 males and 200,000 females died from cancer in Serbia. The overall cancer mortality rate increased nearly equally in both sexes.

GLOBOCAN estimated that there were 8.2 million deaths from cancer in both sexes worldwide in 2012, with an age-standardized mortality rate of 102.4 per 100,000 [2]. The overall cancer mortality in both sexes varies more than three-fold across the world, ranging from about 150.0 per 100,000 in Mongolia, Hungary, Armenia, and Serbia to approximately 50.0 per 100,000 in Namibia and Cape Verde. In males, rates vary more than four-fold, ranging from 209.6 per 100,000 in Armenia to 46.4 per 100,000 in Kuwait. Zimbabwe had the highest cancer mortality rate among females (146.5 per 100,000) and Maldives had the lowest rate (42.3 per 100,000) in 2012. Of 174 countries worldwide, Serbia had the fourth highest cancer mortality rate for both sexes in the same year.

The significant differences in cancer mortality observed between countries could be explained by different prevalence of risk factors; variations in cancer prevention, diagnosis and treatment; the availability and use of high-quality cancer care and resources; and management of health expenditure [18, 19]. In addition, mortality rates partly reflect the varying data quality worldwide [11]. Tobacco smoking is the strongest environmental risk factor known to cause cancer. Tobacco exposure in Serbia is higher than in developed countries [20]. Based on World Health Organization 2013 estimates, the prevalence of tobacco smoking among males was 59.8% in the Russian Federation, 48.7% in China and Mongolia, 44.6% in Serbia, and 24.6% in Finland [20]. In females, the highest prevalence of tobacco smoking was recorded in Serbia (39.7%) and the lowest prevalence was in Bangladesh (0.9%). In 2013, more than one-half (56.3%) of the Serbian population was overweight, classified as pre-obese (35.1%) and obese (21.2%) [21]. Contrary to the overweight category, which included about the same percentage of men and women (20.1 and 22.2%, respectively), the pre-obese category more frequently included men (41.4%) than women (29.1%). According to the 2013 Serbian National Health Survey, conducted by the Institute of Public Health of Serbia, 4.7% of the adult population of Serbia drinks alcohol on a daily basis (8.3% of men and 1.3% of women) [21]. Every other Serbian citizen (54.4%) either does not consume fruit or consumes fruit rarely. Women, more often than men, spent their time at work sitting or standing (48.3% against 38.7%). The share of people aged 65 years and over in the total population of Serbia is 17.4%, indicating an advanced phase of demographic aging [22]. In the period between the censuses of 2002 and 2011, the average age of the entire Serbian population increased by 2 years (from 39.0 to 40.9 years in men and from 41.5 to 43.5 years in women, respectively) [21].

Unfortunately, the prolonged effects of war, manifested in collapsed health care infrastructures, lack of medicines and medical supplies, together with a large number of wounded individuals, created circumstances in which cancer prevention, diagnosis and treatment have been a major challenge in medical practice. One recent meta-analysis pointed out that wars around the globe are making the impact of war-related stress on mortality especially important [23]. However, it is difficult to separate the impact of threats caused by wars on health from the impact of economic sanctions against Serbia, similar to those already seen in Nicaragua and Cuba [24].

According to the 2011 Global Report of the United Nations High Commissioner for Refugees, during the study period, Serbia ranked among the top countries in the world by the number of refugees [25]. The wars in the former Yugoslavia during the 1990s ended with the exile and persecution of many people (around 1,000,000). Twenty years after the first war broke out in the former Yugoslavia, Serbia remains at the top of the list of European countries in terms of forced migration, as well as one of the five countries in the world facing a prolonged refugee crisis [26]. According to the results of the last census in 2011, there are nearly 300,000 forced migrants living in Serbia, equaling 3.9% of the total population. However, neither the age nor sex structure of the refugee population is different from that of the domestic population. Data for refugees were included in the Serbian population in the present study and could not be set apart as a special contingent.

Since the 1980s, mortality rates from all cancers have been falling rapidly for both sexes in North America and many Western European countries [2, 3, 8]. In some countries, however, such as Brazil, Cuba, and Malaysia, the cancer mortality trends have continued to rise in the last decade in both sexes [27]. After two decades of increase, mortality rates for all cancers have been decreasing since 2009 among both males and females in Serbia, similar to its neighbors and many other countries of the former Eastern communist bloc [28]. Reasons for the substantial decline of cancer mortality rates in Serbia since 2009 have not been completely elucidated. Stabilization of the political, social, and economic situation in the country after the 2000s, as well as the implementation of several national guidelines of good practice for the diagnosis and treatment of malignant tumors might, at least partly, explain the observed period effect.

The overall cancer mortality in Serbia can be attributed mainly to mortality from lung to colon cancer in both sexes, prostate cancer in males, and breast and cervical cancer in females. In 2012, Serbia was among the countries with the highest mortality rates from cancers of the lung, colon, pancreas, and brain in both sexes, as well as breast and ovarian cancer in females; other countries with high mortality rates in that year include Hungary, Armenia, Slovakia, Croatia, and Slovenia [2]. Globally, Serbia has the second highest mortality rate from brain cancer for both sexes, after Albania. The high mortality rate owing to brain cancer among women in Serbia is particularly worrisome. On the other hand, mortality rates from cancers of the stomach and prostate are lower in Serbia compared with other European countries.

Lung cancer is the leading cause of cancer death globally. There are different trends of lung cancer mortality in Serbia, mostly reflecting different phases of the smoking epidemic among males and females. These rates in males have recently decreased whereas they have increased in females. The cohort effects observed for both males and females born after 1951 suggest the start of a lung cancer epidemic associated with a high prevalence of tobacco smoking in Serbia. According to data for the population of Belgrade, 49% of males and 25% of females were smokers during 1976–1977, and 51% of males and 37% of females were smokers during 1988–1989 [29].

The Serbian National Health Survey recorded that the overall prevalence of daily smokers was lower in 2013 (29.2%) than in 2000 (33.0%), although a considerable increase was noted in the percentage of daily smokers among women in comparison with 2006 (26.0% in 2013 versus 22.6% in 2006) (21). Thanks to the antismoking campaign that has intensified since 2000 in the country, a decrease in the prevalence of smokers in recent years suggests that the decreasing trend in cancer mortality initiated in 2009 will continue as long as smoking prevalence continues to decline. Our results are in agreement with previous data indicating that international disparities in lung cancer mortality trends most likely reflect different rates of tobacco use in both sexes [30].

The findings of the present study suggest that mortality trends for cancer of the colon, breast, and cervix uteri had already begun to decrease in Serbia before implementation of a national screening program in 2013. The cohort effect has been decreasing for breast cancer mortality in younger generations (those born from 1961 to 1980), indicating that the generative characteristics (such as parity, use of hormonal contraceptives or hormone replacement treatment) and exposures to other risk factors are homogeneous in Serbian females. The use of hormone replacement therapy was very low (less than 1.0%) [31] whereas 1 in 20 women (5.4%) aged 20–49 years used oral contraceptives [21]. In comparison with the previous National Health Survey in 2000, physical inactivity was reduced by 12% in 2006 [21]. The findings of other studies are similar to our results [32].

In Serbia, the noted increase in cancer mortality trends among the oldest birth cohorts (for prostate cancer in males, as well as for pancreatic and hematological malignancies in both sexes) might be attributed to aging and specific exposure to risk factors in early life. The period effects in the mortality trend for hematological malignancies among males only in Serbia are not fully understood. Considering the shorter latency time than for solid organ tumors, the increased mortality trend for hematological malignancies in males during 2006–2015 might be associated with a higher exposure to numerous risk factors during this period, including participation in war and the cleaning and decontamination of fields that were contaminated by various chemicals and other pollutants during bombings [33].

This study revealed an increase in the relative risk for brain cancer mortality in both sexes among the oldest birth cohorts (from 1911 to 1935) in Serbia. This could be attributed to aging and exposure to different environmental factors (especially in agricultural occupations) in cohorts born between the First and Second World Wars, when the Serbian population was mostly rural. In addition, the contribution toward cancer development of exposure to unknown risk factors (such as infectious diseases and so forth) during early life is unclear. Although not significant, the highest increase in mortality for brain cancer was observed in cohorts born from 1946 to 1959, which some researchers have linked with the campaign for eradication of tinea capitis in Serbia during 1950–1959, in which nearly 50,000 children aged 5–15 years were subjected to X-ray treatment of the scalp [34]. The increasing period effects on mortality trends from brain cancer since 1991 could be the result of improved diagnostic procedures. These findings are similar to results reported in other studies [35].

The strength of this study is that it provides the first comprehensive nationwide estimates of cancer mortality in Serbia over a period of 25 years. Another strength is that it covers the entire Serbian population using quality data on cancer mortality, with temporal trends analyzed by both joinpoint and age-period-cohort analysis. However, there were several sources of limitations in this study. We acknowledge that a longer study period might be better for a more accurate assessment of mortality time trends; however, there were no available data for a longer period in Serbia. Of course, there is always a question of whether the cancer mortality trends were a consequence of variations in the process of registering the causes of death, as well as the reliability and validity of death certificates. However, the proportion of cases with uncertain death cause during the study period was non-significantly decreased, so a significant increase in cancer mortality in the observed period can hardly be attributed only to improvement in the quality of mortality statistical data in Serbia. There are no separate data on cancer mortality among refugees, which could possibly confound the cancer mortality pattern in Serbia. Another weakness of this study is related to the inherent limitations of age-period-cohort analysis (such as collinearity among age, period, and cohort effects or ecological fallacy).

## Conclusion

This study represents the first nationwide estimates of cancer mortality in Serbia. The cancer mortality rates place Serbia among the countries with the highest cancer mortality worldwide. Overall cancer mortality has undergone a significant increase in the past decades. The increase in cancer mortality among males during 1991–2009 could be attributed to the increase in mortality from cancers of the lung, colon, and prostate whereas the decrease from 2009 onwards could be attributed to decreased mortality from stomach to lung cancers. In females, the increase in cancer mortality during 1991–2009 could be attributed to the increase in mortality from lung, brain, pancreas, breast, and colon cancers, whereas the decrease in mortality from 2009 onwards could be attributed to decreased mortality from breast to cervical cancers. However, after increasing for two decades, cancer mortality rates in Serbia are finally declining. Nevertheless, the increasing trend in mortality owing to lung cancer among women is particularly worrisome. These rising trends in cancer mortality indicate that improvement in primary and secondary prevention measures is needed.
